# Supplementary material for: Are carbon emissions trading and green financial instruments synergistic? -Comprehensive quantitative research based on content analysis
Source: PLoS One. 2024 Mar 7;19(3):e0298601. doi: 10.1371/journal.pone.0298601 (PMC10919601; doi:10.1371/journal.pone.0298601)
Supplement: S1 Data — (ZIP) [file pone.0298601.s001.zip › Data/地级市Z_F工作报告.zip!/░▓╗╒/╗╞╔╜╩╨/└┤╫╘╣1⁄2╓┌║┼í╛┬φ┐╦╩2╛▌═°í┐_2008.docx]

各位代表：

　　现在，我代表黄山市人民政府，向大会作工作报告，请予审议，并请市政协委员和其他列席人员提出意见。

　　市四届人大一次会议以来的五年，是我市经济社会大跨越发展的五年，是城乡面貌发生深刻变化和人民群众得到更多实惠的五年。五年来，全市人民在省委、省政府和市委的坚强领导下，高举邓小平理论和“三个代表”重要思想的伟大旗帜，认真贯彻党的十六大和十七大精神，全面落实科学发展观，紧紧围绕“一强三大”目标，抢抓机遇，乘势而上，奋力崛起，圆满完成了“十五”计划和市四届人大确定的各项目标任务，开创了我市改革开放和现代化建设的新局面。

　　（一）坚持发展第一要务，经济实力快速提升。积极贯彻宏观调控政策，加快调整经济结构和转变发展方式，促进了经济又好又快发展。地区生产总值从100.5亿元增加到215亿元（预计数，下同），年均增长12.3%。财政收入达20亿元，是2002年的2.5倍。三次产业结构由19.8∶32.4∶47.8调整到14∶37.5∶48.5。坚持以农村经济为基础，扎实推进社会主义新农村建设，大力发展现代农业，农业总产值年均增长4.8%，绿色、有机和无公害农产品认证面积扩大到6.26万公顷。加快推进产业化经营，全市省级以上龙头企业14家，农民专业合作组织达300个，规模以上农产品加工企业年销售收入超20亿元，是2002年的5倍。茶叶年产值突破7亿元，8家企业入选中国茶叶行业百强。坚持以工业经济为支撑，编制发展规划，落实扶持政策，完善服务体系，推进产业集聚、区域集中，工业增加值达60亿元，年均增长15.6%，对经济增长的贡献率由26.5%提高到37%，规模以上工业企业达333户，五年净增199户。累计完成技改投入73亿元，2个产品被评为国家免检产品。园区建设快速起步，全市经济园区累计完成各类投资114亿元，入园企业550户，年工业总产值达47.4亿元。坚持以旅游经济为中心，编制实施规划，强化市场营销，提升产品结构，推进旅游国际化，全市旅游接待量突破1500万人次，旅游总收入突破100亿元，其中，入境游客68.5万人次，创汇1.45亿美元，年均分别增长16.7%、15.3%、16.7%和19.7%。我市成为中国旅游竞争力百强城市，黄山风景区跻身世界地质公园和国家首批5A级景区行列。大力发展现代服务业，认真开展全国服务标准化城市试点工作，全面实施“万村千乡市场工程”，社会消费品零售总额73亿元，年均增长13.7%。金融机构存、贷款余额达253亿元和134亿元，五年分别净增132亿元和66.4亿元。

　　（二）深入推进改革开放，发展活力显著增强。着眼于完善社会主义市场经济体制，顺应经济全球化和区域经济一体化趋势，不断推进体制机制创新，扩大对内对外开放。扎实开展企业改革攻坚，完成401户国有企业改制，盘活资产19亿元，“永新股份”成功上市，“黄山旅游”、“金马股份”完成股权分置改革和定向增发。全面实施农村综合改革、集体林权制度改革和农村税费改革，取消农业税和农业税附加。进一步深化行政管理、社会事业和财政金融体制改革，市城市建设投资公司组建运营，徽商银行在我市开设分行。大力开展全民创业行动，五年新增私营企业3428户，非公有制经济增加值占生产总值的比重达55%，22户民营企业销售收入超亿元。大力实施东向发展战略，积极融入浙江经济带和长三角经济圈，相继开展“对外开放年”、“融入推进年”、“443行动计划推进年”、“招商引资年”和“落实推进年”活动。成功举办4届黄山国际旅游节暨徽文化节，承办第34届环球洲际小姐总决赛、第六届中国国际民间艺术节等重大活动。荣获世界特色魅力城市200强、中国魅力城市、公众最向往的中国城市、中部最佳投资（旅游）城市和浙商（省外）最佳投资城市称号。不断扩大对外交往，与韩国大邱广域市东区等9个城市缔结为友好城市和友好交流城市。开通韩国、日本等国家和地区直航包机，累计1303架次、12.3万人次从黄山口岸进出境。始终把招商引资作为经济工作的重中之重，实施一系列鼓励开放引资政策，全市实际利用外资8925万美元、引进内资108亿元，分别是2002年的8倍和11.7倍。五年累计实现外贸进出口总额4.76亿美元，具有进出口经营权企业由28家增加到280家，外向型经济实现新的突破。

　　（三）大力实施“443”行动计划，城乡面貌焕然一新。坚持规划先行、项目带动，组织实施“443”行动计划，加大投资力度，推进城镇和基础设施建设。全社会固定资产投资达212亿元，五年累计621.2亿元，年均增长41.3%，是建市以来前15年的3.3倍。突出抓好基础设施建设，累计完成交通投入116.65亿元，相继建成徽杭、合铜黄高速公路，黄塔（桃）高速公路、扬绩高速公路绩溪至呈村降段快速推进，乡镇通油路率达100%，行政村通公路率、通水泥（油）路率分别达97.5%和83%；水利、电力、通信设施建设累计完成投资84.6亿元。编制实施城市总体规划，强力推进市中心城区建设，累计完成建设投入143亿元，新建、改建主次干道55条、主要桥梁6座，新增绿地面积48.9万平方米，污水处理厂、垃圾处理场、防洪二期工程、旅游集散中心、城市展示馆等一批基础设施建成运营，基本完成小街小巷小区改造，建成区面积扩大到22平方公里，荣获国家园林城市称号，跻身全国无障碍建设先进城市行列。加大区县城和村镇建设力度，50个建制镇累计完成建设投入90亿元，区县城建成区面积达51.91平方公里，五年扩大了17.55平方公里，17个镇成为全国小城镇建设示范镇、重点镇和环境优美乡镇。扎实推进“抓百村促千村”工程，编制完成全市村庄布点规划和569个村建设、发展规划，累计完成投入近10亿元。全市城镇化率达到38.2%，五年净增9.8个百分点。

　　（四）着力维护群众根本利益，人民生活显著改善。高度重视改善民生，努力提高人民生活水平。全市城镇居民人均可支配收入、农民人均纯收入分别达11350元和4198元，年均增长12.4%和11.6%，连续4年实现两位数增长。城乡居民人均储蓄余额10200元，是2002年末的1.96倍。城乡居民人均住房面积五年净增4.5和6.6平方米。认真落实就业再就业政策，五年新增城镇就业岗位7.6万个，帮助4.45万名下岗失业人员和1.15万名就业困难群体实现再就业，城镇登记失业率控制在4%以内，荣获全省就业再就业工作先进市称号。不断完善社会保障体系，社会保障方面的支出累计达22.1亿元，年均增长32%。全面实施城镇基本养老、医疗、失业、工伤、生育保险制度，实现养老保险市级统筹，企业退休人员养老金比2002年人均月增286元。2千余名未参保集体企业退休人员基本生活得到保障。全市分别有4.75万人和2.03万人参加农村养老保险和被征地农民养老保险。大力推进新型城乡社会救助体系建设，2.6万名城市低保对象实现了动态管理下的应保尽保和分类施保，4.3万农村居民和1.1万五保供养对象获得定期救助。全面落实惠农政策，免除农民政策性负担1.8亿元，近三年直接发放财政各项补贴农民资金4.85亿元，受益农户达36.8万户。扎实开展扶贫开发“整村推进工程”,五年累计投入扶贫资金4.78亿元，实施扶贫开发项目1922个，解决11.7万人口饮水困难，实现户户通电、村村通电话，3个不通车乡公路建成通车，26个省重点地质灾害危险点避让搬迁治理工作基本完成，6.47万人和97个村实现脱贫。累计投资9.4亿元，实施一批为民办实事工程和民生工程，解决城乡居民生产生活中的突出问题，让改革发展成果更多地惠及人民群众。

　　（五）扎实开展精神文明建设，社会事业全面进步。在加快经济发展的同时，更加重视社会事业发展。全市科教文卫体经费支出累计达24.5亿元，年均增长14.9%。坚持用社会主义核心价值体系引领社会思潮，开展社会主义荣辱观教育，不断加大文明创建工作力度，我市和黄山风景区分别获得全省创建文明城市工作先进市、全国文明风景旅游区称号。加强科技创新能力建设，培育国家火炬计划重点高新技术企业4家，实施国家和省级科技项目97个，申请专利355件，组织开展省制造业信息化工程重点城市、科技部数字景区服务示范工程建设。制定实施人才强市战略决定，积极抓好人才交流和高层次人才引进，促进各类人才全面发展。坚持优先发展教育，实现城乡免费义务教育，改造D类危房28万平方米，改建、新建农村寄宿制学校72所，实现国家农村中小学现代远程教育工程全覆盖。职业教育稳步发展，普职招生比连续7年达到1∶1.1以上。黄山学院新校区基本建成，在校生超过万人。编制实施文化大市建设规划，启动徽州文化生态保护实验区申报工作。开展全市第三次文物和非物质文化遗产普查，棠樾村列入中国世界文化遗产预备名单，6个村进入全国历史文化名村行列，全国重点文物保护单位新增7处，6个项目入选国家级非物质文化遗产名录。徽州文化博物馆建设完工。荣获中国徽菜之乡称号。全面实施广播电视村村通工程，档案、地方志和新闻出版、哲学社会科学事业取得新成果。广泛开展全民健身运动，市四运会、首届残运会和黄山国际山地自行车节等品牌赛事成功举办，在省十一运和省残运会、民运会、农运会上取得历史最好成绩。加强公共卫生体系建设，市急救中心和市县疾控中心等建成使用，成功战胜“非典”、禽流感等重大疫情，改造扩建乡镇卫生院53所，新建社区卫生服务机构30个，94.48万农民参加新型农村合作医疗。推进计划生育优质服务创建工作，人口自然增长率控制在4‰以内。扎实开展国家环境保护模范城市创建工作，编制实施生态市建设规划。完成退耕还林4.69万公顷，植树造林3.83万公顷，建成松材线虫病预防体系一期工程。新建农村户用沼气池2.64万口。综合防治水土流失面积250平方公里。大力推进“平安黄山”建设。“四五”普法圆满完成，“五五”普法深入开展。推进基层政权和基层民主政治建设，第六届村委会换届选举工作圆满完成，城市标准化社区和示范社区创建活动扎实有效。强化安全生产监管，完善突发公共事件应急管理体系。建立大信访工作格局，连续10年获得全省信访工作目标管理优秀单位称号。扎实推进社会治安防控体系建设，严厉打击各类违法犯罪活动，跻身全国社会治安综合治理优秀市行列。重视国防动员、人民防空和后备力量建设，被授予全国双拥模范城和全民国防教育先进单位。妇女、未成年人、老年人、残疾人权益得到较好保障。统计、物价、粮食、气象、地震和民族、宗教、对台等工作取得新的成绩。

　　（六）切实转变政府职能，发展环境不断优化。始终把优化发展环境作为政府自身建设的重要内容，大力推进政府管理创新，不断完善工作运行机制。在全省率先并连续四年开展机关效能建设，提高了政府机关的办事效率和服务水平。经过全面清理，减少行政许可和非行政许可项目226项，精简率达44%。着力抓好市行政服务中心建设，全面实行行政审批项目“应进俱进”，扎实推进“两集中、两到位”，现场办结率达到80.7％，窗口办事承诺时限压缩了一半以上。构建“市民连线”公共服务平台，建立了政府、部门与市民、企业在线交流制度，累计受理群众来信来电3.2万件。不断强化政务督查。开展效能、政风和行风建设“三位一体”考核评议，组织市直单位行政“一把手”履职效能监察试点，累计受理各类投诉845件，纠退违规金额2484万元。深入实施行政许可法和依法行政实施纲要，大力推行行政执法责任制。制定发布规范性文件114件。城市管理领域相对集中行政处罚权工作在全省率先推开。坚持民主决策，建立重大事项社会公示、听证制度，获得全国政务公开工作先进单位称号。自觉接受市人大及其常委会的法律监督、工作监督和市政协的民主监督，主动征求和认真听取人大、政协的意见和建议，累计办理人大代表建议和政协提案1587件，满意率达100%。深入开展廉政建设和反腐败斗争，认真落实党风廉政建设责任制，切实加强政府机关思想作风、学风、工作作风、领导作风、干部生活作风建设，加大商业贿赂和损害群众利益不正之风等专项治理力度，加强经济责任审计，严肃查处了一批违法违纪案件。

　　刚刚过去的2007年，全市上下按照“总量上规模，人均争先进”的要求，大力发展旅游、工业和农村经济，强力推进园区、项目、城镇和新农村建设，全市发展速度进一步加快、效益大幅提升、社会更加和谐、民生显著改善。预计地区生产总值、财政收入、社会消费品零售总额分别比上年增长13.2%、27.9%和17.9%，均创十年以来新高；规模以上工业增加值、旅游接待量、全社会固定资产投资分别增长29％、23.3%和30%；引进内资、实际利用外资、外贸进出口总额分别增长39.3%、35.7%和15%；城镇居民人均可支配收入、农民人均纯收入分别增长16.7％和13.2%。经济社会发展实现了新的跨越。

　　各位代表！五年的发展令人振奋，五年的成就来之不易，这是省委、省政府和市委正确领导的结果，是市人大及其常委会和市政协监督支持的结果，是全市人民共同奋斗的结果。在此，我代表市人民政府，向全市广大工人、农民、知识分子、干部、驻黄解放军指战员、武警官兵和政法干警，向各民主党派、各人民团体和其他各界人士，致以崇高的敬意！向关心、支持黄山现代化建设的中央、省驻黄单位和海内外友好人士，向在我市创业的投资者、建设者，表示衷心的感谢！

　　在看到成绩的同时，我们也清醒认识到，我们的工作与人民的期待、与大跨越发展的要求还有不小差距，前进中还面临不少困难和问题，主要是：经济总量偏小、经济结构不优的矛盾仍然突出，转变发展方式的任务十分艰巨；区域、城乡发展不够平衡，中心城市带动力还不强，县域经济相对滞后；就业和社会保障体系还不完善，部分低收入群众生活比较困难，教育卫生、居民住房等方面还存在一些薄弱环节，安全生产、应急管理、生态环保和维护稳定的任务仍然十分繁重。这些问题，归根结底还在于我们发展不足、发展不快。从工作上看，还是我们思想解放和改革开放不够深入，职能转变不能完全适应新形势新任务要求，机关作风和效能建设还不同程度存在一些问题。对此，我们要以对党和人民高度负责的精神，采取切实有效措施，继续认真加以解决，努力把各项工作做得更好。

　　二、今后五年的任务

　　今后五年是我市全面建设小康社会的关键时期。党的十七大胜利召开和科学发展观的深入贯彻，为我们又好又快发展提供了强大动力；国际国内经济持续平稳较快增长和中部崛起战略深入实施，为我们保持良好的发展势头创造了有利环境。特别是我市经过多年的较快发展，积累的能量正加速释放，发展的瓶颈正逐步破解，独特的优势正日益显现。黄山已经站在了大跨越发展的新的历史起点上。当然，对不断增强的资源环境约束、日趋激烈的区域市场竞争、改革发展中的深层矛盾以及各种不确定因素，我们也必须始终保持清醒头脑。要切实增强忧患意识、机遇意识、责任意识，更加自觉地走科学发展道路，不断适应新变化、顺应新期待、实现新要求，奋力开创黄山大跨越发展的新局面。

　　今后五年政府工作的总体要求是：全面贯彻党的十七大精神，深入贯彻落实科学发展观，按照市第四次党代会的总体部署，以推进大跨越发展、建设和谐黄山为主题，以转变发展方式、加快结构调整为主线，以深化改革开放、推进科技创新为动力，以实施“443”行动计划为抓手，加快旅游国际化、新型工业化、农业产业化、城乡一体化进程，更加重视改善民生和促进社会和谐，切实加强节能减排和生态环境保护，促进经济社会又好又快发展，全面完成“十一五”规划，为实现“一强三大”目标和夺取全面建设小康社会新胜利打下更加坚实的基础。

　　按照这一要求，到2012年，全市人均地区生产总值达到29200元，财政收入达到42亿元，城镇化率达到46%，在全省经济发展格局中的位置全面提升；转变发展方式取得重大进展，万元生产总值能耗、主要污染物排放量在完成“十一五”控制性目标的基础上进一步下降，调整经济结构、实现“三个转变”走在全省前列；人民生活水平得到显著提高，城镇居民人均可支配收入、农民人均纯收入分别达到16700元和5900元，覆盖城乡的公共服务体系和社会保障体系基本建立，人人共享发展成果的和谐社会格局初步形成。

　　今后五年的主要任务是：

　　（一）积极发展现代产业体系，推进产业结构优化升级

　　坚持以旅游经济为中心，以推进旅游国际化为主线，按照“保护、开发、完善、提升、创新”的总体要求，全面掀起旅游发展第三次高潮，加快建设“安徽旅游龙头、华东旅游中心、中国旅游名牌、世界旅游胜地”。适应新的消费需求，整合全市旅游资源，推进徽州文化与旅游的深度结合，提升观光旅游，大力发展乡村旅游，积极培育文化体验、休闲度假、商务会展和特种旅游，加快形成一批各具特色、优势互补的旅游经济区，构筑具有国际竞争力的旅游产品体系。适应国际化要求，加快保护、产品、营销、服务、资本、人才与国际接轨，建成一批标志性接待服务设施，推进旅游信息化、服务标准化工程，完善旅游目的地服务体系，打造黄山旅游服务品牌。立足区位、生态优势和产业特点，大力发展教育培训、康体疗养、文化创意、影视娱乐、仓储物流、保险证券等现代服务业，形成黄山特色服务业体系。

　　坚持以工业经济为支撑，按照“三个围绕、一个不上”的要求，继续推进产业集聚、区域集中，走黄山特色新型工业化道路。发挥现有产业优势，坚持存量扩张和增量引进并举，加大骨干企业扶优扶强和中小企业配套发展力度，力争在绿色包装材料、农副产品深加工、机械电子、生物医药等领域打造一批产业集群，年销售收入超亿元企业达30户以上。大力推进信息化与工业化融合，用高新技术和先进实用技术改造提升传统产业，力争五年技改投入超200亿元。着力推进园区建设，创新体制机制，优化发展环境，抓好内引外联，基本建成特色鲜明、优势突出、错位发展的园区体系，夯实工业起跳平台。

　　坚持大力发展现代农业，加强农业基础建设，加快农业结构调整，走黄山特色农业现代化道路。推进区域化布局、规模化生产和产业化经营，做大做强茶产业，大力发展林产业，积极发展蚕桑业、果蔬业、药业、养殖业，构筑现代农业产业体系。着力培育市场主体，力争形成2个超10亿元产业，年营销收入超亿元龙头企业达5家，农民专业合作组织带动农户面达60%以上。大力培养新型农民，加快构建农技推广、动植物疫病防控、农产品质量安全、农村市场等农业服务体系，不断提高农业标准化和信息化、机械化水平。

　　（二）实施项目带动战略，统筹城乡建设发展

　　坚持把项目建设作为经济工作的主抓手、区域发展的硬支撑。全面提升“443”行动计划，围绕建设国际旅游度假基地、全国生态示范基地、华东绿色食品生产加工基地和全省文化产业基地，积极谋划和建设一批战略性、牵动性强的重大产业项目。着力提高基础设施网络化、现代化和一体化水平，突出完善和提升综合交通体系，全面建成覆盖市域的高速公路主骨架，加快构建以皖赣铁路复线为重点的现代化铁路网，扩大机场容量，综合开发新安江航道，奠定全省主要交通枢纽地位。继续加强能源和防洪保安设施建设，基本建成“数字黄山”框架。

　　坚持中心城市与县域互动发展、城镇化与新农村建设协调推进，走黄山特色城镇化道路。加快构建以市中心城区为核心，区县城为骨干，中心镇为纽带的城镇体系，突出抓好市中心城区和市域南部城镇群建设，拉开框架，完善功能，聚集要素，增强辐射，人口规模分别达到25万和50万以上。把壮大县域经济作为统筹城乡发展和推进城镇化的基础性工程，积极鼓励和引导区县立足特色，发挥比较优势，培育主导产业，提升经济实力，加快发展一批综合型、旅游型、工贸型、边界型的建制镇、中心镇和重点镇。建立以工促农、以城带乡长效机制，增加农业农村投入，深入实施“抓百村促千村”工程，加快农村教育、卫生、文化服务和交通、水利等基础设施建设，实现新农村建设新突破。

　　（三）加大改革开放力度，增强经济发展活力

　　坚持推进各项改革攻坚，激活市场主体，完善市场体系，优化市场环境。继续深化国有企业和集体企业改革，加快垄断行业特别是公共服务领域改革，分类推进事业单位改革。大力发展非公有制经济，继续推进全民创业。加快形成统一开放竞争有序的现代市场体系，积极发展各类要素市场和商品市场，大力发展各类社会中介组织。着力加强信用担保和地方金融体系建设，抓好发展和利用资本市场工作，鼓励中小企业、高新技术企业直接上市融资。全面深化农村综合改革，建立农村工作新机制。加快行政管理体制改革，进一步转变职能、理顺关系、优化结构、提高效能，形成权责一致、分工合理、决策科学、执行顺畅、监督有力的行政管理体制，打造服务型政府。

　　坚持把“引进来”和“走出去”更好结合起来，拓展对外开放的广度和深度，提高开放型经济水平。以旅游为依托，办好一批品牌节庆会展活动，实现黄山口岸对外国籍飞机开放，加强对外交流，扩大对外交往，树立开放新形象。以东向发展为突破口，加强区域合作，促进经济融合，成为对接长三角的“桥头堡”和“排头兵”。以招商引资为重点，优化环境，创新方式，引进一批有实力、有品牌、有视野的大企业大集团。以对外贸易为载体，鼓励优势企业加强对外合作，打造一批有国际竞争力的出口生产基地和自主品牌。

　　（四）提高自主创新和节能环保水平，加快转变发展方式

　　坚持把提高自主创新能力作为转变发展方式的中心环节，建设创新型城市。加强科技创业服务，完善鼓励与支持创新的体制机制和政策环境。加快构建以企业为主体、产业为依托、市场为导向、项目为抓手、产学研相结合的区域创新体系，提高集成创新和引进消化吸收再创新能力，推广先进实用技术，实施重大科技专项，力争省级以上工程技术中心和企业研发中心达到16家，全市高新技术企业增加值占规模以上工业比重达30%以上。大力实施知识产权战略和名牌战略。推进人才强市,构建人才资源培训、信息、市场、安居和创业平台，统筹抓好各类人才队伍建设，开创人才辈出、人尽其才的新局面。

　　坚持把节能减排、保护环境作为转变发展方式的重要抓手，以规划为先导、项目为重点、机制为保障，增强可持续发展能力，实现经济发展与生态建设良性互动。大力发展生态经济，抓好生态旅游、生态农业和生态工业，走生态经济化、经济生态化的发展路子。切实保护生态环境，推行清洁生产，加快以节能降耗为重点的技术改造，推进重点领域、行业和企业的资源、能源集约节约利用，认真做好排放总量控制、排污许可和限期治理工作，严格执行环境影响评价及“三同时”制度，抓好循环经济试点，着力防治农业面源污染和城乡生活性污染。加强森林资源保护、水土保持、河道管理等工作，实施一批骨干生态建设工程。全面建设生态文明，积极倡导健康文明、节约资源的消费模式，争创联合国人居环境奖城市，在建设资源节约型和环境友好型社会上迈出新步伐。

　　（五）全面加强文化建设，增强发展的软实力

　　坚持以全面提高人的素质为核心，大力弘扬徽州文化，解放和发展文化生产力，努力把我市建设成为全民素质较高、文化事业繁荣、文化产业发达、文化特色鲜明的地方之一。深入推进徽州文化生态保护实验区建设，重点抓好徽州古建筑古村落保护、徽州文书抢救整理出版、徽州传统工艺传承开发、徽州文化当代价值研究，提高徽州文化保护、研究、开发利用的层次和水平。加快建立覆盖全社会的公共文化服务体系，促进城乡文化协调发展，建设一批重点文化工程和基层文化设施，积极发展新闻出版、广播电视、文学艺术事业，加强文化市场管理，不断丰富群众精神文化生活。深化文化体制改革，强化政策配套，发展文化市场主体，壮大文化工作者队伍，形成一批特色文化产业群，文化产业增加值占生产总值比重提高到9%。广泛开展全民健身运动，努力打造国际体育品牌赛事基地。把社会主义核心价值体系融入国民教育和精神文明建设全过程，深入开展社会主义荣辱观教育，大力倡导“黄山松”和“徽骆驼”精神，广泛开展群众性精神文明创建活动，力争跻身全国创建文明城市工作先进城市行列。

　　（六）切实加强社会建设，努力构建和谐黄山

　　坚持以人为本，更加注重以改善民生为重点的社会建设，努力使全市人民学有所教、劳有所得、病有所医、老有所养、住有所居。进一步扩充提升民生工程，拓展范围、提高标准、完善机制，形成人人共享基本公共服务的和谐局面。优先发展教育，促进义务教育均衡发展，实现高中阶段教育、职业教育和高等教育协调发展，提高教育现代化水平。实施扩大就业的发展战略，健全就业服务体系，完善市场就业机制，实现以创业带动就业，多渠道、多形式扩大就业。深化收入分配制度改革，建立企业职工工资正常增长和支付保障机制，增加城乡居民收入。加快完善社会保障体系，推行农村养老保险和新型农村合作医疗制度，扩大城镇基本养老、医疗、失业、工伤、生育保险覆盖范围。健全社会救助体系，完善城乡居民最低生活保障制度，发展以扶老、助残、救孤、济困为重点的社会福利和慈善事业。继续推进扶贫开发工作。抓好廉租住房和经济适用住房建设管理，解决城镇低收入家庭住房难问题。深化医药卫生体制改革，建立基本医疗卫生制度，提高医技水平和服务质量，基本实现人人享有基本医疗卫生服务目标。加强人口和计划生育工作。推进民主法制建设，全面完成“五五”普法，加快依法治市进程。继续做好国防动员、后备力量建设和双拥工作，巩固发展军政、军民团结。加强基层政权建设，健全基层群众自治机制，推动社区建设上水平、上台阶。加强和改进信访工作，畅通群众依法表达诉求的渠道，强化安全生产和食品药品安全监管，落实突发事件应急管理机制，加强社会治安综合治理，保持社会和谐稳定，力争进入全省首批平安市行列。

　　三、2008年的工作

　　2008年是全面贯彻落实党的十七大精神的第一年，也是实施“十一五”规划承上启下的关键一年。做好今年各项工作，意义重大。我们要认真贯彻党的十七大、中央和省经济工作会议精神，按照市委四届五次全会确立的指导思想和作出的总体部署，全面落实“六个坚持”原则，准确把握加强和改善宏观调控的政策导向，在坚持好字优先的前提下，立足于赶，着眼于超，努力实现科学发展、和谐发展、率先发展。

　　今年经济社会发展的主要预期目标是：地区生产总值增长13%以上；财政收入增长20%；全社会固定资产投资增长25%；引进内资增长20%，实际利用外资增长15%；旅游接待量增长18%，旅游总收入增长30%；全社会工业增加值增长19%以上；社会消费品零售总额增长16%，外贸进出口总额增长16%；城镇居民人均可支配收入增长8%，农民人均纯收入增长7%，居民消费价格指数涨幅控制在4.5%以内；城镇新增就业岗位1.47万个，城镇登记失业率控制在4%以内；人口自然增长率控制在4.6‰以内；万元生产总值能耗下降4%；城镇化率提高1.4个百分点。

　　为此，抓好以下十个方面的工作：

　　（一）深入开展社会主义新农村建设

　　加快发展现代农业。围绕发展壮大主导产业，优化区域布局，抓好基地建设，实施生态茶园“双十”带动工程，发展果蔬、贡菊、养殖和花卉苗木基地，新增毛竹、蚕桑、油茶基地1184公顷。积极实施农业产业化提升行动，加大龙头企业引进和培育力度，力争新增营销收入超5000万元龙头企业2家、农民专业合作组织30个。着力提高农产品品质，推进农业标准化生产，建设农产品质量检测中心，力争新增绿色、有机和无公害农产品认证面积6000公顷，树立黄山优质生态农产品形象。加大品牌建设和市场营销力度，办好茶叶暨名优农产品展示交易会，推出一批名牌农产品。推进农科教结合，实施农业科技入户工程，建设科技示范乡镇、示范村，抓好科技特派员基层创业行动试点。

　　改善农村生产生活条件。全面落实各项强农惠农政策。继续组织实施新农村建设规划，推进村庄综合整治、建设，100个示范村率先达到“十有”标准，150个村实现基本达标。重点抓好农田水利基本建设，解决9万人安全饮水问题，建成1000口抗旱灌溉机井，完成33座病险水库除险加固任务，提高农业抵御自然风险能力。改造通村公路500公里，全市94%行政村通水泥（油）路。继续改善农村通信条件，抓好农业信息化进村入户和广播电视村村通工程，提高远程教育设施利用水平。培训农村劳动力1万人次，打造特色劳务品牌。

　　坚持把发展壮大县域经济作为新农村建设的重要途径，认真落实加快县域经济发展的实施意见，从规划引导、组织管理、要素保障等方面细化措施，解决制约县域经济发展的薄弱环节，形成综合配套的发展环境。充分发挥区县的主体作用，全面增强县域自主发展能力，以项目为支撑、以开放为动力，做大经济总量、壮大财政实力、增加农民收入，加快工业化、城镇化步伐。进一步完善考核评价体系，加大奖惩力度，促进县域经济快速跃升，在全省县域发展中争先进位。

　　（二）全力推进旅游国际化

　　打造国际化旅游产品。推进黄山景区世界旅游精品建设，提升和强化龙头带动作用。实施中法乡村旅游合作示范项目，培育国际乡村旅游精品，力争西递、宏村进入5A级景区行列。挖掘徽州文化资源，包装文化旅游项目，推出大型旅游演出活动，形成文化旅游新亮点。加大产品整合力度，开发一批新的旅游线路，提升全市旅游产品整体竞争力。加快太平湖、雨润、奇墅湖度假区（村）和茶博园、新徽天地、新安江山水画廊等项目建设，增强旅游发展后劲。

　　推进国际化旅游营销。运用媒体、展会和节庆活动等多种手段，加大黄山旅游形象和产品的宣传推广。努力增开、加密国际国内航线，拓展日韩、东南亚市场和国内中远程市场。办好奥运会火炬接力传递活动，扩大奥运旅游市场。加强国际合作，实施借力营销，开拓欧美等境外远程市场。依托合铜黄和徽杭高速公路，适应国家实施新休假制度，积极培育淡季、非周末和中短程市场。继续强化整体联动营销机制，发挥企业主体作用，增强市场开拓合力。

　　加快建设国际化旅游目的地。抓好服务标准化城市试点，推进旅游“六要素”服务标准化体系建设。启动旅游信息化试点城市项目，完成数字景区服务示范工程，建设营销、服务、管理一体化的旅游信息化网络。加强适应个性化旅游需要的配套设施建设，强化旅游集散中心功能，扩大旅游公交网络，构建自驾游服务体系。延伸旅游产业链条，大力发展旅游商品生产，创新发展徽菜产业。加强旅游市场监管，提高从业人员素质，营造全社会服务旅游的良好氛围。

　　（三）促进工业提速增效

　　着力培育产业集群。编制实施产业集群发展规划，力争在绿色包装材料、食品和医药等产业率先取得突破。把扶优扶强与培育集群结合起来，进一步加大政策支持力度，促进30户骨干企业加快裂变扩张，亿元以上企业突破20户。积极培育中小企业，提高产业配套能力，新增规模以上企业40户。强化工业运行监测和协调服务，帮助企业加强管理，提高运行质量和效益。做好企业用工对接工作，加大技工培训力度。支持企业争创著名商标和名牌、免检产品。

　　大力发展园区经济。进一步加强基础设施建设，完善管理体制和运行机制，提升综合服务水平，为园区加快发展打下坚实的基础。继续加大园区招商和项目建设力度，努力实现建成项目早见效，在建项目早完工，签约项目早建设，不断提高园区发展的质量和效益。加强对全市园区产业发展的规划引导，促进各园区有序、集约发展具有比较优势的特色产业。力争全市园区完成投资52.5亿元、实现工业增加值16.4亿元、税收收入2亿元，其中黄山经济开发区分别达到10亿元、1.6亿元和3000万元。

　　努力提高自主创新能力。引导企业加大研发投入，做大做强省高新技术产业基地，争取新建1～2个省级以上工程技术研究中心。积极发展科技企业孵化器，促进科技成果产业化。继续组织实施制造业信息化等重大科技专项，着力抓好一批重点技改项目，技改投入超30亿元，高新技术产业营业总收入增长27%以上。继续完善、落实人才政策，激发现有人才创新创造活力，发挥黄山籍在外人士作用，办好留学人员黄山创业园，鼓励引资与引智、引进与引用相结合，确保人才引得进、留得住、用得好。

　　（四）抓好城镇和基础设施建设

　　加快重点项目建设。继续推进“443”行动计划，着力实施468个市级重点项目。突出抓好大交通建设。力争黄塔（桃）高速公路年底建成通车，开工黄祁高速公路，加快推进扬绩高速公路绩溪至呈村降段和国省道改建工程。新建黄山机场国际候机楼。建立健全项目推进机制，统筹抓好环境评价、土地报批、节能评估、安全准入等前置环节，落实项目建设条件，提高项目工作效率。集中力量加快皖赣铁路复线、黄杭快速铁路、铜黄金铁路、新安江流域综合开发、黄千高速和扬绩高速公路黄山区连接线等一批重大项目的前期工作，新增项目储备300亿元以上。积极对上争取，力争更多项目得到国家和省里支持。密切跟踪、主动配合做好国家和省主体功能区规划编制工作。

　　不断提高城镇建设水平。抓好市中心城区建设，完成投资42亿元。推进主次干道连网，加快停车和公交设施建设，启动实施天然气工程，改建供排水系统。加快江南旧城改造和新城建设，推进屯光大道、齐云大道片区开发，建成游泳馆、全民健身中心。抓好“城中村”综合改造和重要街道、节点的风貌整治，完成南滨江景观带建设，提升绿化、亮化和美化水平。编制实施市域南部城镇群总体规划和近期建设规划。抓好区县城建设。开展建制镇新一轮规划修编和集镇总体规划编制工作，力争50个建制镇完成投资30亿元。推进重点景区周边乡镇与景区一体化发展，支持城郊型村镇率先构建城乡一体的公共服务设施体系。提高规划设计水平，加大保徽、建徽、改徽力度，彰显徽派城镇特色。抓好数字化城市管理示范工程，继续推进公用事业市场化改革，推行建设工程有效最低价中标制度，加强质量管理，提高建设管理水平。

　　　（五）认真做好财政金融工作

　　不断完善公共财政体系。建立健全稳定的财政增收机制，积极培植支柱财源，提高财政收入质量，增加可用财力。加强和改进支出管理，优化支出结构，继续深化部门预算、国库集中收付、政府采购和非税收支改革，加强对预算执行的法律监督和资金使用的绩效评价，提高资金使用效益，集中更多的财力投向社会建设和公共服务领域。认真做好事业单位规范津补贴工作。完善国有资产运营监管，组建市国有资产运营公司。做好财政投资评审工作，建立地方政府偿债准备金，加强政府债务控制和管理。

　　努力缓解融资瓶颈。适应从紧货币政策新形势，着力改善金融生态环境，推进企业信用制度建设，建立中小企业贷款风险补偿机制，鼓励金融产品创新，加强银企项目对接，力争全年信贷投放增幅超全省平均水平。积极发展地方性金融机构，加快农村金融体系建设，争取建立1～2家农村合作银行。壮大现有担保机构实力，积极发展民营担保组织。进一步整合城市国有资产，做强市城市建设投资公司，扩大与国家开发银行的合作。积极争取外国政府和国际金融组织贷款。推进企业直接融资工作，引导上市公司申报公司债券试点和加大再融资力度，力争1～2户企业进入上市辅导期或发行企业债券。

　　（六）加快推进改革开放

　　　进一步加大改革力度。深化政府机构改革，加快转变政府职能，推进政企分开、政资分开、政事分开、政府与市场中介组织分开，强化社会管理和公共服务，切实把政府经济管理职能转到主要为市场主体服务和创造良好发展环境上来。深入推进农村综合改革，实现集体林权制度改革阶段性目标。以聘用制为重点，稳步实施事业单位人事制度改革。继续深化企业改革，认真解决改制企业遗留问题。着力推进公平准入，完善服务体系，建立健全有利于全民创业的体制机制，培育一大批创业主体。坚持并完善政府与民营企业家联系制度，推进民营企业制度创新和科技创新，提高整体素质，壮大企业家队伍，努力造就新一代徽商。

　　扩大对外交流与合作。强力推进招商引资，更加注重方式方法创新，提高招商引资成效。认真做好项目谋划，增强招商针对性，充分调动各方面招商引资的积极性，发挥专业招商队伍的作用，力争在优势产业招商、重大项目招商上取得新突破。加快转变外贸增长方式，优化进出口结构，办好公用保税仓库，促进加工贸易转型升级，鼓励重大技术装备和资源性产品进口。进一步加大东向发展力度，加快融入杭州都市圈、浙江经济带和长三角经济圈，推进与毗邻地区的互动发展。积极做好外事侨务和台湾事务工作，扩大国际交流合作，发展友城关系。全力办好2008中国国际徽商大会、第十二届中国黄山国际旅游节暨徽文化节等重大活动。

　　（七）加大生态建设和环境保护力度

　　扎实推进节能减排和资源节约利用。编制实施节能、节水、资源综合利用等重点专项规划，出台促进循环经济发展配套政策。抓好全市第一次污染源普查。落实节能减排工作责任制，加快实施一批治污、节能重点工程，抓好化工、建材等重点行业和重点企业节能减排，淘汰落后生产能力。建立政府强制采购节能产品制度。推广节能省地环保型建筑，建设节水型城市。加强农业生产性污染治理，继续实施生态家园富民工程，新建沼气池4000口。启动循环经济示范试点，加快完善城市生活垃圾和废旧物资回收、加工、利用体系。抓好土地利用总体规划修编，开展城乡建设用地增减挂钩试点工作，落实建设用地投资强度和容积率“双控”标准，全面清理闲置土地，推进土地节约集约利用。

　　切实保护生态环境。加强风景名胜区、自然保护区、森林公园和地质公园的保护与管理，推进齐云山申报世界自然遗产和歙县清凉峰申报国家自然保护区工作。巩固退耕还林、荒山造林、封山育林成果，抓好林政管理和森林防火工作，争取实施松材线虫病预防体系二期工程。落实建设项目生态恢复措施，推进高速公路沿线景观绿化整治，加强河道管理和饮用水源地保护，治理水土流失面积105平方公里。推进新安江流域生态补偿机制试点，加大月潭水库前期工作力度。继续开展创建环境优美乡镇、生态文明村和生态示范户活动。综合整治城市油烟、噪声扰民问题，开展绿色社区、绿色单位、绿色家庭创建活动。力争进入国家环保模范城市和中国人居环境奖城市行列。

　　（八）积极发展各项社会事业

　　坚持优先发展教育。继续实施义务教育经费保障机制改革，认真落实高校和中职学校学生资助政策。全面完成在册D类危房改造任务，加快乡镇寄宿制学校或九年一贯制学校建设，认真推进素质教育和新课程改革。加快高中教育普及进程，大力发展中等职业教育。积极整合和引进教育资源，加快筹建高等职业学院和技师学院。支持黄山学院做好迎接本科教学水平评估工作，为地方经济和社会发展服务。完成黄山电大扩建，发展成人教育和社区教育。

　　加强医疗卫生服务。完善疾病预防控制、基本医疗服务和卫生执法监督体系，建成一批标准化乡镇卫生院、村卫生室和规范化社区卫生服务机构。加强医疗卫生队伍和医德医风建设，规范医疗服务行为，为群众提供安全、有效、方便、价廉的医疗卫生服务。积极支持新安医学和中医药事业发展。抓好食品药品和餐饮卫生监管。加强工业企业劳动卫生管理，提高职工自我防护意识。大力开展爱国卫生运动，争创国家卫生城市。着力提升计划生育技术服务水平，完善农村部分计划生育家庭奖励扶助制度，扎实推进关爱女孩和综合治理出生人口性别比工作，继续加强城市和流动人口管理，稳定低生育水平，提高出生人口素质。

　　大力发展文化事业和文化产业。推进徽州文化生态保护实验区建设。继续抓好文物和非物质文化遗产普查、申报和保护工作，公布一批市级文物保护单位和非物质文化遗产名录，推进徽州文书抢救整理出版工作，探索试行古民居认领保护，争取皖南古村落千村保护与发展项目取得新进展。加强乡镇综合文化站、“农家书屋”和社区文化基础设施建设，大力弘扬民俗民间文化，广泛开展群众性文化活动，加强网吧、棋牌室管理。推进文化体制改革，发展文化产业，命名一批市级文化产业示范基地，争取屯溪老街跻身国家级文化产业示范基地。切实抓好徽州文化研究、文艺创作、文化创意和非物质文化遗产传承人队伍建设。积极开展全民健身运动，举办市五运会，认真备战省十二运，继续办好山地自行车节、健走节、登山节和“黄山论剑”等赛事活动。启动市广播电视节目上701台转播工作。出版《黄山市志》。开工建设市档案馆。徽州文化博物馆、黄山报业大楼、市美术馆和歌舞剧团、徽剧研究中心工程建成投入使用。抓好群众性精神文明创建活动，力争进入全省文明城市行列。

　　（九）切实保障和改善民生

　　努力促进就业再就业。进一步落实就业再就业优惠政策，做好职业介绍、技能培训和就业指导等服务工作。继续抓好创业园区和创业街建设，支持自主创业和自谋职业。完善面向所有困难群众的就业援助制度，消除城镇“零就业”家庭，实现“4050”人员再就业1450人。统筹做好高校毕业生、进城务工人员、被征地农民就业和退役军人安置工作。认真贯彻劳动合同法，规范和协调劳动关系，落实最低工资标准，健全农民工工资保障机制，强化劳动保障监察执法和争议调处工作，维护劳动者权益。

　　不断完善社会保障体系。加大社会保险扩面征缴力度，城镇基本养老、医疗、失业保险参保人数分别达到9.7万、26.7万、9.7万人。提高企业退休人员养老金水平。建立健全农村养老保险制度，加快推进被征地农民基本养老保险工作，提高新型农村合作医疗补助标准并实现全市覆盖。进一步完善城乡社会救助体系，继续提高城乡最低生活保障和农村五保供养标准，健全重大传染病病人医疗救治和生活救助保障机制，逐步提高城乡医疗救助水平。加大深山区、库区和老区扶贫开发力度，做好大中型水库移民后期扶持工作。发展残疾人事业，继续做好白内障患者复明、精神病患者医疗救助和农村贫困残疾人危房改造工作。加强老龄工作，关爱农村留守儿童，建设市老年大学和青少年活动中心。建成廉租住房1.66万平方米，对人均住房建筑面积低于10平方米的城市低保户实行应保尽保。抓好蔬菜、生猪等副食品生产流通和粮食储备工作，加强价格监测和市场监管，及时落实对低收入群众的补助。加大民生工程实施力度，在继续抓好18项民生工程的基础上，再实施一批省市民生工程，不断提高人民群众受益面和受益度。

　　努力提高社会管理水平。深入开展城市标准化社区和示范社区创建活动，完成第七届村委会换届选举，推进村务公开和民主管理示范单位创建工作。支持工会、共青团、妇联等人民团体参与社会管理和公共服务。增强全民国防观念，加强民兵预备役建设，做好拥军优抚安置工作，巩固和发展全国双拥模范城创建成果。加强民族宗教工作。强化安全生产责任制，抓好隐患排查整改和重大危险源监控，严防重特大事故发生。完善应急管理体系，加快应急指挥平台建设，切实做好突发性公共事件预防与应急处理工作。完成重点地质灾害危险点避让搬迁治理。落实信访工作责任制，加强人民调解组织建设，及时排查调处矛盾纠纷，积极预防和妥善处理群体性事件。加强公安基层基础工作，健全社会治安防控体系，防范和打击违法犯罪活动，做好刑释解教人员安置帮教和社区矫正工作，推进“平安黄山”建设迈上新台阶。

　　（十）加强政府自身建设

　　新形势新任务对政府工作提出了新的更高的要求。我们一定要认真贯彻党的十七大精神，把科学发展观贯彻落实到政府工作的各个方面，增强科学发展意识，提高科学发展能力，创新科学发展机制，创造科学发展业绩。

　　进一步提高政府创新力。紧跟时代发展潮流，大兴学习之风，切实加强与公务员岗位职责相适应的基本理论、基本知识、基本技能培训，准确把握中国特色社会主义理论体系的科学内涵和精神实质，全面提高在工业化、信息化、城镇化、市场化、国际化深入发展背景下分析、解决问题的能力。弘扬求真务实精神，大兴调查研究之风，针对经济社会发展的一些薄弱环节和事关全局的重大问题，深入开展专题调研，超前抓好战略谋划，增强工作的前瞻性、针对性和创造性。发挥群众首创精神，建立和完善有利于广泛集中民智的决策机制，推行重大事项公开咨询、听证和公示制度，加大人民建议征集力度，提高决策透明度、公众参与度，推进决策的科学化和民主化。

　　进一步提高政府执行力。强化发展意识、大局意识、宗旨意识，敢于负责、善于协调、勤于落实，以振奋的精神和良好的作风推进各项工作。下气力减少开会发文和检查评比，腾出更多的时间和精力抓大事、干实事、办好事。加强政府领导班子建设，严格执行民主集中制，更好地发挥核心保证和表率带动作用。加大机构整合力度，合理配置人力资源，精简和规范各类议事协调机构及其办事机构，健全部门间协调配合机制。优化政府工作流程，精简审批事项和办事程序，加快发展电子政务，继续抓好市县（区）行政服务中心建设，巩固和推进城市管理、旅游等领域相对集中行政处罚权工作。建立和完善体现科学发展的政府绩效考核体系，改善和加强目标管理、监察督查，落实行政首长负责制和行政问责制，确保令行禁止、政令畅通。坚持不懈推进机关效能建设，深入开展效能、政风和行风“三位一体”评议以及“百家企业评机关”活动，优化经济发展环境，努力实现“办事效率最高、服务质量最好、工作节奏最快、商务成本最低、社会环境最优”的目标。

　　进一步提高政府公信力。完善制约和监督机制，让权力在阳光下运行，确保权力正确行使，打造诚信政府、法治政府和廉洁政府。扩大政务公开的渠道和范围，推进公用事业单位的办事公开。继续办好“市民连线”和“政风行风热线”，强化群众和舆论监督。自觉接受人大及其常委会的法律监督、工作监督和政协的民主监督。贯彻依法行政实施纲要，强化行政执法监督，严格落实行政执法责任制和责任追究制，不断提高政府依法行政水平。扎实推进惩治和预防腐败体系建设，全面推进经济责任审计工作，坚持用制度管权、管事、管人，严格执行领导干部廉洁自律各项规定，坚决纠正损害群众利益的不正之风，严肃查处各类违纪违法案件，以廉政建设的实际成效取信于民。

　　各位代表！我们已经从新的历史起点出发，踏上新的征程。让我们高举中国特色社会主义伟大旗帜，紧密团结在以胡锦涛同志为总书记的党中央周围，在中共黄山市委的领导下，进一步解放思想，开拓进取，扎实工作，夺取全面建设小康社会的新胜利，谱写黄山人民美好生活的新篇章！
